# Perceptions of scheduled vs. unscheduled directly observed visits in an internal medicine residency outpatient clinic

**DOI:** 10.1186/s12909-020-1968-1

**Published:** 2020-03-04

**Authors:** Joanna Rea, Christopher Stephenson, Emily Leasure, Brianna Vaa, Andrew Halvorsen, Jill Huber, Sara Bonnes, Luke Hafdahl, Jason Post, Majken Wingo

**Affiliations:** Mayo Clinic, 200 First Street, SW, Rochester, MN 55902 USA

**Keywords:** Direct observation, Internal medicine, Resident assessment, Feedback, Camera, Clinical practice, Outpatient clinic, Formative assessment, Learning

## Abstract

**Introduction:**

Learners may subconsciously change their behavior once they know they are being observed, and this Hawthorne effect should be considered when designing assessments of learner behavior. While there is a growing body of literature to suggest direct observation is the ideal standard for formative assessment, the best method to directly observe learners is unknown. We explored scheduled and unscheduled methods of direct observation among internal medicine residents in the outpatient continuity clinic to advance the understanding of both observation methods.

**Methods:**

We conducted a thematic analysis of faculty and internal medicine residents in an outpatient clinic setting. A semi-structured interview guide for focus group sessions was created. Focus groups were used to explore the internal medicine resident and core teaching faculty perceptions of the scheduled and unscheduled direct observation methods in the outpatient clinc. An experienced qualitative research interviewer external to the internal medicine residency was moderating the sessions. Eight peer focus groups were held. Abstraction of themes from focus group transcripts identified resident and faculty perceptions of the different observation methods.

**Results:**

Focus groups had 14 resident participants and 14 faculty participants. Unscheduled observations were felt to be more authentic than scheduled observations since residents perceived their behavior to be unmodified. Unscheduled observations allowed for increased numbers of observations per resident, which permitted more frequent formative assessments. Residents and faculty preferred remote video observation compared to in-room observation. Participants found direct observation a useful learning tool for high-yield, specific feedback.

**Conclusions:**

Unscheduled remote direct observation captures authentic clinical encounters while minimizing learner behavior modification. An unscheduled observation approach results in more frequent formative assessment and therefore in more instances of valuable feedback compared to scheduled observations. These findings can help guide the best practice approaches to direct clinical observation in order to enhance residents learning and experience.

## Background

Faculty educators are increasingly encouraged to use more direct observation of authentic clinical work to assess the skills of learners [1]. A growing body of literature suggests direct observation as the foundation and ideal standard for formative assessment, but to what extent a scheduled observation and/or in-room observer modifies those assessments is unknown [1–5]. The Hawthorne effect describes a change in behavior in response to observation and assessment [6]. Within medical education, the Hawthorne effect is relevant while learners are observed during patient care as their behavior may be consciously or subconsciously altered. A method of observation that can reduce the behavior modification while assessing learners through direct observation may be the ideal tool.

Milestone-based assessments are a key component of clinical performance evaluation, and direct observation is critical for valid assessment of many milestones [3, 7–9]. The Accreditation Council for Graduate Medical Education Common Program Requirements for internal medicine (IM) residency requires that competencies be assessed via direct observation in both the inpatient and outpatient setting [10]. Inpatient skill assessment may lend itself more naturally to direct observation during bedside rounds with patients, family, and teams, [11] but providing effective and reliable observations in the outpatient clinic can be a challenge for many IM residency programs. Barriers in outpatient clinic include faculty time constraints, competing demands for faculty, financial disincentives, clinic space, and clinic structure/function barriers [2, 4, 12, 13]. Additionally, residents do not usually request to be observed as direct observation can be perceived to threaten resident autonomy, efficiency and interfere with resident-patient relationship [4]. Much of the current medical literature on direct observation represents hospital-based assessments or procedural/surgical assessments [11, 14]. There is a paucity of data on best methodology to perform direct observation in the outpatient setting. While direct observation can be accomplished in various methods (in room, via a one-way mirror, via camera), the optimal direct observation method in outpatient clinic is not known.

In this study, we sought to explore resident and faculty perceptions of two different methods of direct observation (scheduled vs. unscheduled) in IM outpatient clinic. A direct observation method that would allow supervising faculty to observe learners in a less invasive, more authentic manner could begin to mitigate the Hawthorne effect and might result in more productive feedback, learning and evaluation.

## Methods

### Setting

Our research was conducted at Mayo Clinic in Rochester, Minnesota. Our IM program consisted of 148 residents and 50 core teaching faculty in outpatient continuity clinic during this study. The core teaching faculty worked closely with resident learners throughout all 3 years of IM training. IM resident outpatient clinic was split between two locations that provide comprehensive care to patients seen in a typical suburban clinic. Two thirds of residents had outpatient clinic in the division of CIM (Community Internal Medicine) while one third had clinic in the division of GIM (General Internal Medicine). Residents were assigned to either the CIM or GIM clinic at the start of their intern year. This assignment was done as part of the normal residency operations and was not part of our study design. Demographics of the resident learners and the core teaching faculty were similar. Both clinics had the same faculty to resident ratios. Residents had the same number of patients scheduled per half day based on their post-graduate year. Our institution has a decades long experience with direct observation of IM residents in outpatient clinic.

#### Scheduled observation visits

The direct observation method in the GIM outpatient clinic utilized scheduled direct observation visits. Patients were slated into a designated “observation” appointment. All parties (resident, faculty, and patient) knew an observation was occurring 100% of the time. The patient was roomed in an exam room equipped with a camera. Patient consent for observation was obtained by the resident prior to the start of the visit. The video from this camera was real-time, live feed (not recorded), and the video feed could be viewed by the observing teaching faculty from an adjacent private room. Headphones or computer speakers were used for audio. If there were barriers to using the camera (e.g. camera or viewing screen malfunction), an in-room observation could occur by the supervising teaching faculty who was known to the resident learner and whose purpose was for observing the resident learner. The faculty would choose whether they would observe in the room or via remote camera based on resident and faculty preferences and whether the camera system was operational. Approximately 4 observations per IM resident were scheduled per academic year. The faculty would typically observe the entire encounter. Of these scheduled observation visits, what proportion were observed in the room vs. via camera was not tracked. Verbal feedback to the learner occurred after the visit and an electronic evaluation was submitted by the core teaching faculty member who observed the encounter.

#### Unscheduled observation visits

The direct observation method in the CIM outpatient clinic utilized unscheduled direct observation visits. Patients were put into the usual appointment slots for each resident and roomed into a usual exam room. Each exam room had a camera. With the unscheduled observations, only remote camera observation was utilized. No in-room observations occurred. When patients were roomed, consent for camera activation was obtained by a licensed practical nurse. If patients agreed, the camera was turned on and the rooming nurse left the room. The video feed was real-time and not recorded. The video feed was observed remotely via a designated private computer monitor with a selectable video tile screen. This monitor was in a group work room with other core teaching faculty and residents in the same continuity clinic, but the work station was cordoned off with privacy screens. Audio from the camera was heard through headphones. The faculty then selected which encounter to observe from the video tile screen as there were often many possible encounters to observe. There was no assignment of the encounter, resident or patient in advance and therefore, direct observation was unscheduled. Faculty could choose to observe the whole encounter or a portion of the encounter. The resident knew that with each patient, an observed visit was possible. However, the resident was unaware if the visit was being observed or not. On average, approximately 9 observations per IM resident per academic year occurred with unscheduled observations. The length of observation was not tracked. Verbal feedback to the learner occurred after the visit and an electronic evaluation was submitted by the core teaching faculty that observed the encounter.

### Data collection and analysis

Focus groups explored the IM resident and core teaching faculty perceptions of the direct observation methods used in the outpatient clinic setting during the academic year 2017–2018.

The data collection period was from October to December 2018. All IM residents and outpatient clinic core teaching faculty were given the opportunity to participate in focus groups. After approval from the Mayo Clinic’s internal review board, the Principal Investigator (JR) recruited residents and faculty by email. Two emails were sent to recruit participants without individual solicitation by the Principal Investigator. Written informed consent was obtained by all participants. Focus groups included representatives from all resident levels and all career stages of core teaching faculty.

A semi-structured interview guide was created with assistance from a group of qualitative research experts and was iteratively revised until consensus.(Appendix) Peer focus groups were conducted to assess perceptions of directly observed visits; such methods have been used to capture similar perceptions in the outpatient setting in previous research [15]. A total of eight 60-min focus groups were conducted by the same moderator. There were two focus groups held for each population (scheduled observation residents, scheduled observation faculty, unscheduled observation residents and unscheduled observation faculty) with 3–4 participants in each group.

Data was collected with full audio transcripts of each focus group. Data was analyzed by a qualitative researcher with 24 years of experience external to Mayo Clinic. The researcher’s experience and background were unlikely to influence the interpretation of results. An exploratory thematic analysis was performed as described by Guest et al. [16] Given the exploratory nature of our question, transcripts were analyzed inductively and a codebook was developed [17]. After coding, common themes were developed through an iterative process which included discussion, meetings and memos. No new themes emerged in the final focus groups indicating saturation of themes. Thematic analysis was corroborated by principal investigator (JR). Three of the authors participated in faculty focus groups for the observation method they experienced. They were not introduced as authors; they neither guided the discussion nor influenced other participants.

Threats to validity were assessed using a previously published framework for critically appraising qualitative research [16, 18]. To ensure fidelity of assessments, an experienced qualitative research interviewer external to the institution and the IM residency was used as the moderator. The use of multiple data analyzers, peer debriefing of themes and insights, and member checking was employed to support the reliability of data. Transcripts were provided and reviewed (Tables 1, 2 and 3) to support validity for confirmability and objectivity.

## Results

A total of 28 participants were included in the focus groups: 6 scheduled observation residents, 6 scheduled observation faculty, 8 unscheduled observation residents and 8 unscheduled observation faculty. Review of focus group transcription abstracted several themes.

### Theme #1 scheduled vs unscheduled observations

Both resident groups (scheduled and unscheduled) were asked if they preferred knowing or not knowing about the observation if given a choice. Unscheduled observation participants preferred unscheduled visits. Unscheduled observation residents thought their work with patients was more authentic with unscheduled visits and their behavior was more genuine. Unscheduled observation residents preferred not knowing and stated that having spontaneously observed visits helped their growth because “it forces you to bring your A game all the time,” “it is a truer situation and what they observe is more realistic” and its “more organic.” (Table 1) Unscheduled observation faculty also preferred the unscheduled observations as they could get “a lot more real sense of what business as usual was,” and “it’s not as contrived,” and “we are really seeing them doing what it is that they do.”

**Table 1:** Scheduled vs. Unscheduled observations

Scheduled Observation-Residents	Unscheduled Observation-Residents
• “If I had to choose, I would choose knowing ahead of time but with the understanding that you do lose a lot when you do know … because you might change how you act.” • “I think scheduled is definitely more comfortable, but I do see the advantage of not having scheduled observations because, inherently, we are all prone to that bias. When someone’s observing you, you are going to behave differently.” • “It [scheduled observation] is more comfortable and I focus on things that I think I need to work on and have them specifically observe those skills, which allows me to walk away from that encounter with more learning from it.” • “When it is scheduled, I actively invest in what I hope to walk away from that experience with.”	• “If you don’t know, it’s a truer situation and what they observe is more realistic.” • “I prefer not knowing about an observation.” • “My preference is towards the not knowing or not having it scheduled.” • “I do recognize that not knowing probably is more organic and gives me better feedback.” • “I think not knowing enhances the ability for you to get more honest feedback if you don’t know when you’re being observed, I guess would be the biggest positive aspect to it. Because oftentimes when we’re doing something wrong, we don’t know that we’re doing it wrong. It’s not like we’re inherently doing it wrong on purpose, so I think that’s the best positive aspect.” • “I think you learn more by having someone see what you actually do in clinic versus what you’re showing them you can do. I think it’s more valuable to have them see what you do when you’re not on the stage.” • “I think you learn more in that setting because people are observing you in your natural habitat versus you putting on a show. You learn more because it makes you change what your actual habits are versus your habits when you’re being observed.”
Scheduled Observation-Faculty	Unscheduled Observation-Faculty
• “I would prefer the residents to know about the observation.” • “Scheduled observation makes it a point of having them self-reflect on what they want advice or feedback on, then I think that provides a greater, richer opportunity for learning than unscheduled.” • “I think it [scheduled observation] probably does affect the resident performance. When you know you’re going to be watched, everybody does things a little more thoroughly.”	• “I prefer unscheduled when residents don’t know if they’re being observed, so we really are seeing them doing what it is they do.” • “Unscheduled is not as contrived.” • “I prefer unscheduled as some residents, if they know they’re being observed, will put forth a different effort which can be quite painful to watch.”

Scheduled observation residents stated they behave differently when they know they are being observed and they might change how they act. However, if given a choice, scheduled observation residents preferred scheduled observations as they could prepare for the observation and demonstrate their skills. With scheduled visits, they also had the opportunity to identify learning goals in advance since they knew they were being observed. Scheduled observation residents felt this had clear impact on their learning. Scheduled observation faculty noted that scheduled observation likely affects the resident performance. However, scheduled observation faculty liked that scheduled observed visits allowed residents to discuss the case with faculty before the visit and target a specific area of learning they are seeking, which is seen as a benefit and positively impacts their development.

### Theme #2 preference regarding observation modality (remote camera versus in-room)

All residents in the scheduled and unscheduled groups preferred remote camera observation compared to in-room observation. (Table 2) The scheduled observation residents identified that having faculty in the room was uncomfortable, a “barrier to communication,” and made the flow of the visit “more difficult.” Other descriptions from scheduled and unscheduled residents regarding faculty in the room were “awkward,” “uncomfortable,” “strange,” and “loss of credibility.” Overall, both groups of residents preferred observation via camera.

**Table 2:** Preference regarding observation modality (remote camera versus in-room)

Scheduled Observation-Residents	Unscheduled Observation-Residents
• “Mostly [remote camera] just because it ensures a one-on-one encounter with the patient so that the two of you are working together and not in between some intermediate source.” • “It’s [remote camera] more of the natural environment” • “So remote, to me, feels more like a real patient encounter and is a more natural kind of environment. Someone sitting in the room with you is kind of babysitting.” • “It [in-room] makes you feel like your autonomy has been taken away” • “I think it [remote camera] also enhances your clinical decision making. You know you’re the only one in the room and you know you have to make some sort of medical decision while you’re there instead of kind of leaning or using a crutch of someone being in the room with you.”	• “It changes the entire encounter when the faculty’s with you and you lose all credibility.” • “Remote video is better, because if you have someone there, it just impacts your patient interaction. The patient will keep looking at whoever is, like, older, seems more prestigious in the room. They won’t think that I’m their doctor.” • “Plus, they’d [faculty] have to just totally keep their mouth shut … which wouldn’t happen.” • “I mean, if we’re trying to become physicians and our place of work, and art and everything that we do and love and put everything into this vocation of ours is supposed to be in that room with us and the patient, this is a time that we learn how to do that and navigate that without somebody else sitting in the room. And I think that’s a super special and sacred place, and to learn how to navigate that now is primarily why we’re here training, so I think it really undermines that.”
Scheduled Observation-Faculty	Unscheduled Observation-Faculty
• “If the goal is to teach them to think on their own and decide on their own, then they need to be in the room alone with the patient.” • “If I am in the room, the patient looks to me then as the physician, because I’m the highest-ranking physician. You don’t get that at all with the remote, right, so there’s no looking past the resident to the attending, “Oh, what do you think?” kind of thing.”	• “We want our residents to establish rapport with their patients, develop longitudinal relationships. They can’t do that with someone staring over their shoulder” • “Video observation allows you as the faculty to have more flexibility as a preceptor. If you’re observing something but then there is a bolus of residents who need staffing, you can temporarily step away. So, it gives you a little bit more of finger on the pulse of how things are going otherwise. If you’re pulled into the room, you’re out of commission for that complete duration of time.”

Both scheduled and unscheduled faculty groups perceived remote observation as less intimidating, less anxiety-provoking for the residents, helped avoid undermining the resident, and is overall less contrived. Unscheduled observation faculty remarked that remote observation provides another advantage by allowing them the ability to simultaneously observe the encounter, review the patient’s chart, and examine resident documentation. It also provides more teaching flexibility for the faculty by allowing them to step away should they be needed by other learners.

### Theme #3 perceptions regarding learning and feedback

Unscheduled observation residents found the feedback especially helpful and informative since they were engaging in authentic clinic work and were less aware of being observed. (Table 3) These residents thought the feedback provided after an observed visit allowed for specific examples of what they did well or what could have been improved upon and were the most impactful to their learning compared to feedback after an unobserved visit.

**Table 3:** Perceptions regarding learning and feedback

Scheduled Observation-Residents	Unscheduled Observation-Residents
• “Overall, it’s been positive … the feedback we get from the observing faculty is way more specific than the feedback we would get if were just to present the patient.” • “I think observation definitely affects my learning. I can think of five specific things that I do now because of the feedback that I received during observed encounters.” • “I derived the most learning from having my physical exam observed.” • “So having an observer during challenging patient conversations really helped with my communications skills.” • “Scheduled observed visits adds efficiency when you are staffing cases. Then you’re afforded time to get to some of the more polishing details and advanced principles.” • “When they [faculty] already have heard the entire history, you can jump straight to the impression, report and plan and spend more time discussing differentials and what you would do and follow-up, rather than going back to the history and starting from scratch.”	• “I think the type of feedback, the quality of feedback from the preceptors given both verbally as well as written is more helpful and more direct, more specific when someone has directly observed me than some of the more generic comments that come when you just staff cases in the clinic and weren’t observed.” • “Specific examples in real time are far more educational.” • “Direct observation is better for learning exam skills because they can watch and correct you.” • “I feel like some of the best kind of timely, specific feedback that I’ve gotten has been as I am discussing a patient with a preceptor who just observed me in that encounter.” • “I’ve found feedback useful in many ways. Getting feedback on the art of medicine and how to communicate better has been very pivotal for my training.”
Scheduled Observation-Faculty	Unscheduled Observation-Faculty
• “When we are not observing, the evaluation and feedback is an assessment of their knowledge verses observation allows more feedback about skills and attitudes.” • “Observation allows us to give feedback on the how, not the what.”	• “Feedback after observation is much more specific. It’s much more behavioral. It’s what I saw, it’s what you said, it’s how you said it, it’s all that sort of stuff we wouldn’t capture otherwise.” • “Observation allows us to give feedback on the attitudes. Attitudes are important.”

Scheduled observation residents also found the feedback from direct observation impacted their learning and shaped the way they handle patient encounters. The positive reinforcement on observed behaviors was a common aspect of the feedback provided. Scheduled observation residents also thought that observation speeds up presenting a patient encounter and allows more time for individualized learning. With the time saved, there is more time for “discussing differentials and what you would do and follow-up,” “get to some of the more polishing details,” or “go straight to decision making.” Scheduled observation faculty stated that when a resident presents a case to them in clinic, the evaluation and feedback is an assessment of their knowledge. However, when a resident is directly observed, targeted feedback on exam skills and attitudes; the “how you are doing it” versus the “what you are doing” can be provided.

## Discussion

This study confirms the importance of direct observation and highlights some advantages and constraints of two different types of direct observation. Unscheduled observation seems to demonstrate one significant advantage compared to scheduled observations: it allows the resident learner to perform authentic clinic work with the patient while minimizing behavior modification seen with scheduled observation. This is seen as a benefit by the resident learners and the teaching faculty. This suggests that when the learner is less mindful of being observed, the assessment is potentially more valid. Sound assessments from faculty are more productive when learners are engaging in authentic clinical work yet remain less aware of the observation. Therefore, minimizing the Hawthorne effect with unscheduled direct observation may be beneficial for both the learner and the faculty observer.

With unscheduled observations, faculty can perform a greater number of observations per resident compared to scheduled observations. Faculty can watch partial visits. This allows for additional opportunities for feedback because a faculty member may be able to observe, for example, the physical exam portion of the appointment for one resident and the counseling portion for a different resident. This permits more frequent assessments of resident behaviors with increased occasions for formative feedback. In addition, partially observing visits allows faculty to be readily available to staff other learners’ patients in a busy clinic day. Therefore, this set-up may not require an increase in the faculty time or resources needed to complete observed visits.

Another finding was the nearly unanimous preference for direct observation via remote camera instead of in-room observation from residents and teaching faculty. To our knowledge, demonstration of such a strong remote camera observation preference has not been described prior. Most of the available literature on direct observation in an education setting involves in-room observations in the hospital, in a procedural setting, or in a clinic setting [11, 19]. It is accepted that any observation is better than no observation with regard to identifying learner behaviors and attitudes that are otherwise hard to assess [20]. However, there is no data to suggest that in room observation is superior to camera observation. This study suggests that with the use of high fidelity cameras and audiovisual equipment, there is no reason to believe that in room observation would gather more information than with the use of high fidelity remote camera observation.

Overall, residents felt that direct observation was a useful learning tool regardless of whether it was scheduled or unscheduled. Direct observation had a positive impact on their education specifically due to detailed, high-yield feedback. Specific feedback helped them continue certain behaviors and consider change for others. Many residents also noted how direct observation saves time when presenting patients since they might not have to report the whole history, exam, or background of the patient’s narrative and can use that time to learn the finer teaching points.

In our era of milestone and competency-based education, we suggest that direct observation is critical and necessary to accurately assess our learner’s development. Direct observation methods and systems like the ones described in this study can facilitate these goals. Both methods of observation in this study had aspects that were beneficial. It is important to consider which method of direct observation is best for an individual program as a helpful tool for behavior-based milestone assessment.

Our study has several strengths. The focus group moderator was an experienced researcher who conducted all of the sessions and was external to our institution, minimizing potential for bias. Themes generated by analysis of the transcripts were verified by both the qualitative researcher and the principal investigator. The semi-structured interview format allowed for exploration of additional themes if identified during the focus groups. Faculty were not present for resident focus groups, which reduced desirability bias by limiting faculty influence on resident responses.

There are several limitations to our study. Our research was conducted when the resident continuity clinic merged into a single location that is currently utilizing one method of direct observation (unscheduled remote observation visits; merger July 1, 2018). Scheduled observation participants were asked to recall perceptions from 6 or more months when the scheduled direct observation visits were occurring. These participants were initially prompted not to be influenced by their experiences in the merged clinic to ascertain perceptions regarding scheduled observation, but confounding was possible. Additionally, a few of the authors participated in the faculty focus groups for the observation method they experienced. These authors were not introduced and did not shape the conversation. However, the presence of these authors could have led to social desirability bias affecting the results. Our academic program has a large number of residents and faculty preceptors which may limit generalizability to other IM residency programs with smaller environments or fewer faculty to facilitate observed visits. This study also only looked at direct observation in the outpatient setting and therefore limits transferability to the inpatient domains of IM training. Finally, this study was based on the perceptions shared during the focus groups and therefore, any ideas or perceptions not shared remain unidentified.

## Conclusions

Direct observation is essential for IM residents in outpatient clinic to help reinforce and coach behaviors that are necessary to becoming competent clinicians. Both types of direct observation methods in this study have advantages and challenges. Unscheduled remote direct observation captures authentic, organic clinical encounters with learners while minimizing behavior modification. Unscheduled remote observation visits also allow for higher number of observations to assess resident performance and offers increased opportunities for feedback. At our institution, direct observation via remote camera is preferred over in-room observation by both residents and faculty. Feedback from direct observation (whether scheduled or unscheduled) was highly valued by resident learners. These findings may help guide best practices for outpatient education clinics to enhance resident assessment and skill development.

## Data Availability

The full transcript from all focus groups was the dataset used and analyzed during the current study. This dataset is available from the corresponding author on reasonable request.

## Appendix

### Interview Guide

#### Residents

**Table:**

**GIM Residents:** The purpose of this study is to understand your experience regarding direct observation in the GIM resident clinic for the 2017–2018 academic year. The system used in the GIM resident clinic for the 2017–2018 academic year involved a scheduled patient visit with observation either in the room or utilizing a camera. The observed visit with the resident learner and the patient was clearly identified in advance of the observation. We understand that this observation system is no longer in use. However, for the purposes of this discussion, please set aside your experience with the current system and respond only regarding the observation system used in the GIM resident clinic for the 2017–2018 academic year. This is very important.

**Table:**

**CIM Residents:** The purpose of this study is to understand your experience regarding direct observation in the CIM resident clinic for the 2017–2018 academic year. The system used in the CIM resident clinic for the 2017–2018 academic year involved a remote video system observation. The visit observed was not identified in advance either by the learner or by the faculty member. The observation was unscheduled and the decision to observe was made by the preceptor/faculty at the time of the visit	

1. Overall, what are your thoughts about this type of observation?

2. If you had to choose, would you say this type of observation is positive or negative? Why?

3. Does this type of observation impact your learning in any way? If yes, how?

4. How would you describe the feedback you received during or after these observations?

5. Did the feedback impact your learning in any way? If so, how?

6. Would you say you were comfortable or uncomfortable with this type of observation overall? Why is that?

7. If you had a choice between direct in the room observation vs. remote video observation, which would you prefer? Why?

8. If you had a choice between knowing you are going to be observed or not knowing, which would you prefer? Why?

9. Was discussing goals and objectives with your staff prior to your observation a part of the process? If so, what were the benefits or drawbacks of this part of the observation process?

10. Does knowing or not knowing if you are going to be observed impact your performance in any way? If so, how?

11. Does knowing or not knowing if you are going to be observed impact your learning in any way? If so, how?

12. If you could change the way in which these observations are done, what would you change?

#### Faculty

**Table:**

**GIM Faculty:** The purpose of this study is to understand your experience regarding direct observation in the GIM resident clinic for the 2017–2018 academic year. The system used in the GIM resident clinic for the 2017–2018 academic year involved a scheduled patient visit with observation either in the room or utilizing a camera. The observed visit with the resident learner and the patient was clearly identified in advance of the observation. We understand that this observation system is no longer in use. However, for the purposes of this discussion, please set aside your experience with the current system and respond only regarding the observation system used in the GIM resident clinic for the 2017–2018 academic year. This is very important.

**Table:**

**CIM Faculty:** The purpose of this study is to understand your experience regarding direct observation in the CIM resident clinic for the 2017–2018 academic year. The system used in the CIM resident clinic for the 2017–2018 academic year involved a remote video system observation. The visit observed was not identified in advance either by the learner or by the faculty member. The observation was unscheduled and the decision to observe was made by the preceptor/faculty at the time of the visit.	

1. Overall, what are your thoughts about this type of observation?

2. In relation to completing milestone evaluations, what are your thoughts about this type of observation? Useful or not useful?

3. Does this type of observation impact learning in any way? If yes, how?

4. How would you describe the feedback provided during or after these observations?

5. Did the feedback impact learning in any way? If so, how?

6. Was discussing goals and objectives with residents prior to observations a part of the process? If so, what were the benefits or drawbacks of this part of the observation process?

7. Does a resident knowing or not knowing whether they are going to be observed or not impact their performance in any way? If so, how?

8. Does a resident knowing or not knowing whether they are going to be observed or not impact their learning in any way? If so, how?

9. If you could change the way in which these observations are done, what would you change?

***********************************************************

*LP* 10. Would you say you were comfortable or uncomfortable with this type of observation overall? Why is that?

*LP* 11. If you had a choice between direct in the room observation vs. remote video observation, which would you prefer? Why?

*LP* 12. If you had a choice between residents knowing they are going to be observed or not knowing, which would you prefer? Why?

*LP* 13. If you had to choose, would you say this type of observation is positive or negative? Why?

*LP = Lower priority. These questions will only be asked by the moderator if time allows.*

## Abbreviations

CIM: Community internal medicine
GIM: General internal medicine
IM: Internal medicine
